# Synaptically Induced Long-Term Modulation of Electrical Coupling in the Inferior Olive

**DOI:** 10.1016/j.neuron.2014.01.005

**Published:** 2014-03-19

**Authors:** Alexandre Mathy, Beverley A. Clark, Michael Häusser

**Affiliations:** 1 Wolfson Institute for Biomedical Research and Department of Neuroscience, Physiology and Pharmacology, University College London, Gower Street, London WC1E 6BT, UK

## Abstract

Electrical coupling mediated by gap junctions is widespread in the mammalian CNS, and the interplay between chemical and electrical synapses on the millisecond timescale is crucial for determining patterns of synchrony in many neural circuits. Here we show that activation of glutamatergic synapses drives long-term depression of electrical coupling between neurons of the inferior olive. We demonstrate that this plasticity is not triggered by postsynaptic spiking alone and that it requires calcium entry following synaptic NMDA receptor activation. These results reveal that glutamatergic synapses can instruct plasticity at electrical synapses, providing a means for excitatory inputs to homeostatically regulate the long-term dynamics of microzones in olivocerebellar circuits.

## Highlights

• Chemical synapses trigger long-term depression of inferior olive electrical coupling
• Depression of electrical coupling requires NMDAR activation and calcium entry
• Plasticity is not triggered by postsynaptic spiking alone and EPSPs remain unchanged
• Excitatory inputs can thus homeostatically regulate synchrony patterns in the olive

## Teaser

The interplay between electrical and chemical synapses is crucial for determining patterns of synchrony in neuronal networks. Recording in the inferior olive, Mathy et al. show that coincident excitatory synaptic input and spiking can cause a sustained suppression of electrical coupling.

## Introduction

Interest in electrical synapses between neurons in the mammalian brain has seen a dramatic resurgence in the past decade with the recognition that gap junction-mediated coupling is both widespread and cell type specific in adult mammalian neural circuits (Bennett and Zukin, 2004; Connors and Long, 2004; Hestrin and Galarreta, 2005). The importance of electrical coupling in regulating synchronous activity in neuronal populations has been extensively explored using both experimental and modeling approaches (Manor et al., 1997; Deans et al., 2001; Bartos et al., 2002; Leznik and Llinás, 2005; Blenkinsop and Lang, 2006; De Gruijl et al., 2012). Given that electrically connected neurons also receive chemical synaptic input, a growing body of work has demonstrated that the interplay between electrical and chemical synapses is crucial for determining patterns of synchrony on the millisecond timescale not only in the inferior olive (Lang et al., 1996; Marshall and Lang, 2009; Wise et al., 2010) but also in neocortical (Gibson et al., 1999; Beierlein et al., 2000) and cerebellar (Vervaeke et al., 2010) interneuron networks. Moreover, there is good evidence that chemical neurotransmitters can modulate the strength of electrical synapses. The potential importance of such a transsynaptic interaction between chemical and electrical synapses is underlined by the close anatomical proximity between the two synapse types observed in the “mixed” electrical and chemical synapses found in many parts of the nervous system (Sloper and Powell, 1978; Rash et al., 1996). Thus far, most attention has been focused on short-term interactions between electrical and chemical synapses on the millisecond timescale, but it has long been speculated that chemical synapses may also drive long-term regulation of mammalian electrical synapses (Connors and Long, 2004). Electrical synapses can undergo long-term modification by spiking activity (Haas et al., 2011), metabotropic neurotransmitters (Landisman and Connors, 2005; Mills and Massey, 1995), and dopaminergic activation (Kothmann et al., 2009). While experiments in goldfish Mauthner cells have shown that activation of fast glutamatergic synapses can produce long-term potentiation of neighboring electrical synapses (Yang et al., 1990; Pereda and Faber, 1996; Smith and Pereda, 2003), whether chemical synaptic input can also regulate the strength of mammalian electrical synapses in the long term remains unclear.

The electrically coupled neurons of the inferior olive represent a particularly attractive system for attacking this question. Electrical coupling is mediated by Connexin 36 gap junctions formed between dendritic spines of neighboring olivary cells (Long et al., 2002). Importantly, the gap junctions are located in close proximity to glutamatergic and GABAergic synapses housed within a glomerular structure (Llinas et al., 1974; Sotelo et al., 1974; de Zeeuw et al., 1989, 1990a, 1990b): an anatomical arrangement that provides an excellent substrate for intersynaptic interaction. While fast, short-term modulation of olivary electrical coupling by both GABAergic and glutamatergic synaptic inputs has long been suggested (Llinás, 1974; De Zeeuw et al., 1998; Lang, 2002; Jacobson et al., 2008; Hoge et al., 2011; Bazzigaluppi et al., 2012), no direct evidence exists to support long-term modulation of electrical synapses in the mammalian inferior olive. Here we have addressed this question by performing simultaneous patch-clamp recordings between coupled neighboring olivary neurons. We demonstrate robust, long-term depression of electrical coupling triggered by physiological patterns of glutamatergic synaptic input.

## Results

### Synaptic Stimulation of Olivary Neurons Depresses Electrical Coupling

We obtained simultaneous recordings from electrically coupled pairs of olivary neurons in the principal olive and medial accessory olives in rat brainstem slices. Electrical coupling was measured by injecting negative current pulses into either cell (Devor and Yarom, 2002; Landisman and Connors, 2005) and assessing the resulting voltage change in the noninjected cell. Coupling was detected between almost all neighboring neurons (with a coupling probability higher than 80%). The mean coupling coefficient was 0.014 ± 0.001 (measured at a resting potential of −54.2 ± 0.4 mV; input resistance 32.7 ± 0.6 MΩ, n = 111 pairs). We then activated excitatory synaptic input to the neurons and increased stimulus strength until the evoked excitatory postsynaptic potentials (EPSPs) reliably induced spiking (spiking probability averaged over both cells ≥0.5). For each pair, we recorded the electrical coupling by alternating delivery of negative current pulses to each cell for a baseline period. This was followed by an induction protocol consisting of 50 synaptic stimuli at 1 Hz, with steady-state depolarizing current such that the neurons fired the characteristic burst of spikes observed in olivary neurons in response to sensory stimulation in vivo (Chorev et al., 2007; Khosrovani et al., 2007).

**Figure 1:**
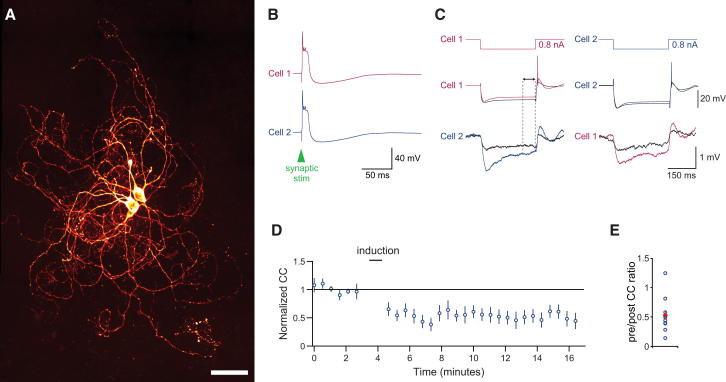
Long-Term Depression of Electrical Coupling between Inferior Olivary Neurons Triggered by Activation of Chemical Synapses (A) Confocal image of two electrically coupled olivary cells filled with biocytin and processed with streptavidin-Alexa Fluor 488 (scale bar, 50 μm). (B) One of 50 synaptically evoked spikes, triggered in both cells at 1 Hz, used as the induction protocol. (C) Average responses of a pair of cells to alternate current injections into cell 1 (left) and cell 2 (right; colored traces, preinduction; black traces, postinduction). Mean voltage deflection in the injected cell (middle traces) and in the coupled cell (bottom traces) were measured over the interval shown (arrowheads and dashed lines). Induction caused depression of electrical coupling between the cells as measured by negative current injections. (D) Time course of the coupling coefficient (n = 10 pairs; error bars represent SEM). (E) Population data for the ratio of normalized coupling coefficients after and before induction; the coupling coefficient was reduced by 47% ± 10% (p = 1.1 × 10^−6^).

Following the induction protocol, the average coupling coefficient was significantly lower in nine out of ten pairs of connected cells (Figure 1; average reduction of 47% ± 9.9% from an initial coupling coefficient of 0.012 ± 0.003; p < 1 × 10^−5^, n = 10 pairs). This depression of coupling was sustained for more than 15 min, with the longest recordings showing plasticity 25 min after induction. Consistent with the reduction in coupling, input resistance was also increased following induction (mean input resistance 38.4 ± 18 MΩ after induction; 21.8% ± 7.5% increase; p < 0.01). Only small changes were seen in resting membrane potential (6% ± 3% hyperpolarization, n = 20 cells, p = 0.067) and sag ratio (decrease by 13.9% ± 4.5%, n = 20 cells, p = 0.054) following induction.

Changes in coupling can result from either changes in input resistance or junctional conductance, and this conductance can be estimated indirectly by combining transfer resistances and input resistance. Using this estimate of gap junctional conductance confirms that coupling remains significantly reduced after induction (48% ± 12% reduction; p < 1 × 10^−4^; Figure S1A available online). Experiments were also performed in voltage clamp to provide a more direct readout of coupling (Figures S1B–S1D). Cells were held at −55 mV during the baseline and postinduction period. For plasticity induction, synaptic stimuli were paired with short (10 ms), 10 mV depolarizations to allow the cells to fire bursts of unclamped spikes. After induction, coupling was significantly reduced (by 15% ± 1% of control; p < 0.01, n = 7 cells), consistent with our current-clamp experiments.

Finally, we tested the effect on coupling coefficient of higher-frequency olivary spiking in the presence of more intense synaptic input. Spikes at 4 Hz, evoked by depolarizing current pulses, were paired with 25 Hz bursts of synaptic input timed to provide synaptic glutamate release throughout the postsynaptic spike (Figure S2A). This “theta”-like activity was designed to mimic pairing protocols that are typically used to induce synaptic plasticity in other brain areas. We used 100 pairings, yielding a comparable number of spikes to the maximum number used for the 1 Hz pairing protocol. Although a small increase in coupling coefficient was seen in two out of five pairs, on average there was no significant difference from baseline in postinduction coupling coefficient with this protocol (Figures S2B–S2D; 2.7% ± 17% increase; n = 5 cells, p = 0.625).

### Mechanism of Electrical Coupling Plasticity

**Figure 2:**
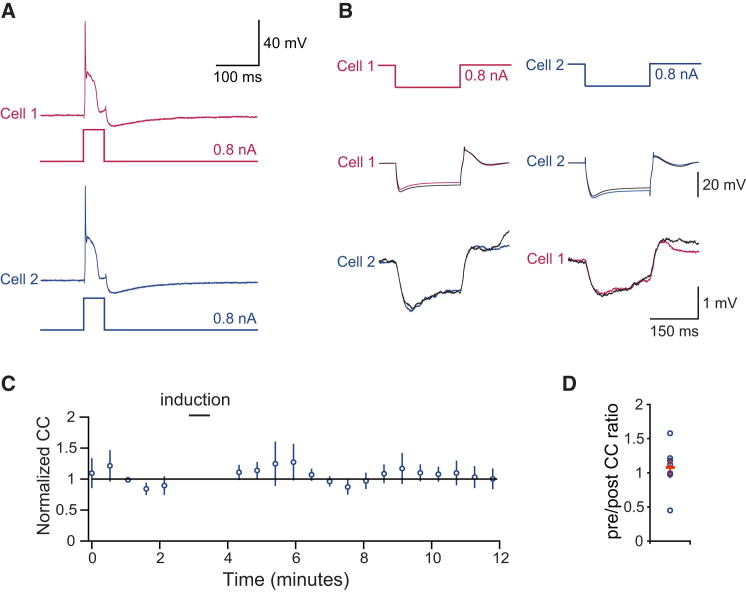
Spiking Alone Is Insufficient to Trigger Plasticity of Coupling (A) Induction consisted of 50 spikes in both cells triggered by current injections. (B) Average responses to alternate current injections in a representative paired recording before and after the induction protocol. (C and D) Time course of normalized coupling coefficient (n = 7 pairs) (C) and ratio of normalized coupling coefficients after and before induction (D). Mean (red bar) and SEM are indicated.

We next investigated the conditions required for the induction of the depression of coupling. To test whether olivary bursting on its own could change the coupling, we used an induction protocol that consisted of steady-state depolarization and 50 brief current pulses (30 ms, 800 pA) at 1 Hz, which reliably triggered the olivary burst in all of the cells (Figure 2). In contrast to synaptic stimulation, this protocol produced a small but statistically insignificant increase in coupling (by 7% ± 13%; p = 0.42; n = 7 pairs). We also performed experiments where plasticity induction was carried out in the absence of spiking (Figure S3). In this case, induction consisted of 50 synaptic stimuli at 1 Hz with no accompanying depolarizing current pulses, which resulted in depression of coupling (32% ± 13% depression after baseline, n = 7 pairs, p < 0.05). Therefore, action potential bursts alone are insufficient to produce changes in coupling strength, and action potentials are not required for triggering plasticity with synaptic stimulation.

To rule out the contribution of GABAergic synaptic mechanisms to the plasticity, we repeated the induction protocol in the presence of blockers of GABAergic synaptic transmission (10 μM SR-95531 and 2 μM CGP-55845 to block GABA_A_ and GABA_B_ receptors, respectively). Under these conditions, the plasticity protocol also produced a significant long-term depression of coupling (27% ± 9.8% of control; n = 15, p < 0.001; Figures S4A and S4B). This demonstrates that plasticity induction does not rely on GABAergic synaptic transmission.

**Figure 3:**
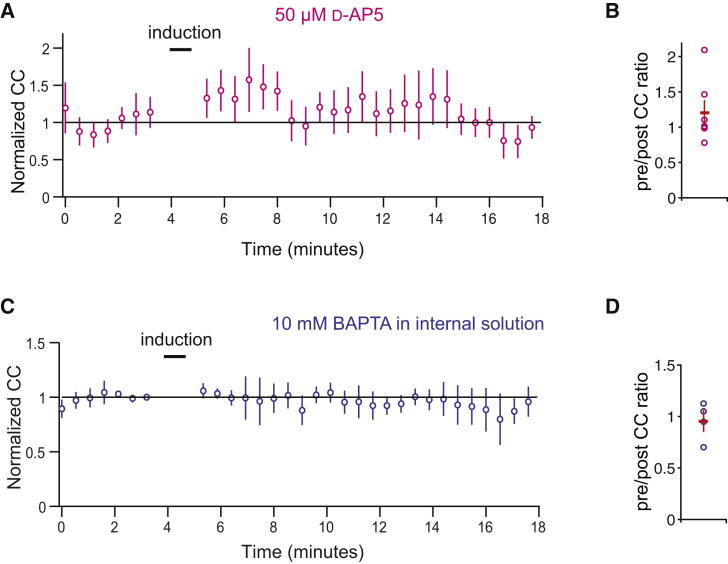
Plasticity of Coupling Depends on NMDA Receptor Activation and Calcium Entry (A and B) Plasticity induction (protocol as in Figure 1) in the presence of D-AP5 (50 μM) prevents changes in the coupling coefficient (A). The population data are shown in (B) (n = 7 pairs; p = 0.093). (C and D) Plasticity induction (protocol as in Figure 1) with 10 mM BAPTA in the internal solution prevents changes in the coupling coefficient (C). The population data are shown in (D) (n = 4 pairs; p = 0.49).

Since olivary neurons express NMDA receptors in the vicinity of gap junctions (Hoge et al., 2011), and interactions between NMDA and connexin molecules have been seen in other systems (Yang et al., 1990; Pereda and Faber, 1996; Pereda et al., 1998; Smith and Pereda, 2003), we investigated whether NMDA receptor activation is necessary for the plasticity of electrical coupling. Using the same induction protocol as for Figure 1 in the presence of D-AP5 (50 μM), a potent NMDA receptor antagonist (Figures 3A and 3B), no significant depression of electrical coupling was observed following induction (coupling after induction 120% ± 18% of baseline, p = 0.093; n = 7 pairs).

We hypothesized that calcium entry, through NMDA receptors and other sources, was responsible for the depression of coupling, and therefore repeated the experiment in the absence of D-AP5 but with 10 mM BAPTA (Figures 3C and 3D), a high-affinity calcium chelator, in the internal solution. This prevented any change in coupling by the induction protocol (coupling after induction 95% ± 10% of baseline, p = 0.49; n = 4 pairs).

What are the downstream effectors of NMDA-receptor-mediated calcium entry? One possible candidate is CaMKII, which is known to be activated by NMDA-receptor-driven calcium entry (Lisman et al., 2002) and which is present close to olivary Connexin 36 plaques in the inferior olive (Alev et al., 2008). We therefore repeated the plasticity experiments with KN62, a selective CaMKII inhibitor (Tokumitsu et al., 1990), in the pipette solution (10 μM). No significant plasticity was observed under these conditions (coupling after induction 89% ± 29% of baseline; n = 4 pairs, p = 0.70; Figures S4C and S4D).

### Plasticity Induction Is Specific to Electrical Coupling without Affecting Chemical Synapses

**Figure 4:**
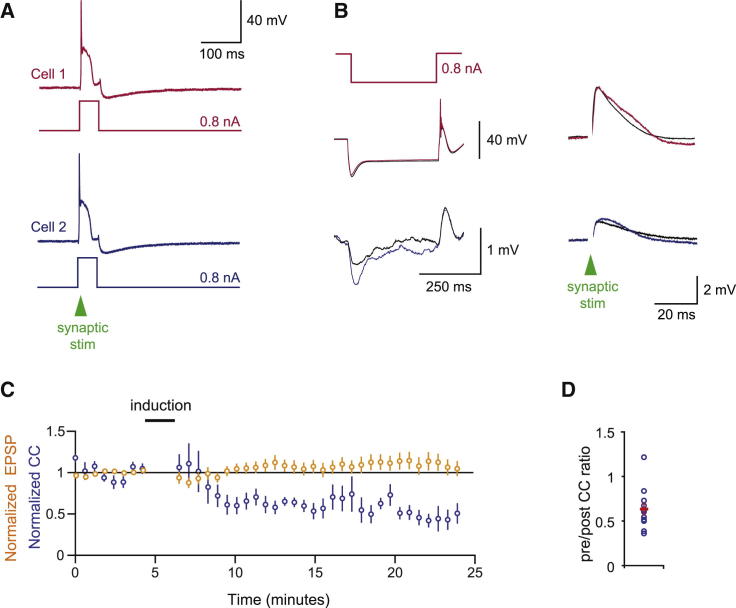
Plasticity Induction Affects Electrical but Not Chemical Synapse (A) Induction: lower intensity stimulation and current pulses were combined to cause spiking and synaptic responses (100 pairings at 1 Hz). (B) Sample pair: coupling was depressed (left traces) after pairing, but the synaptic response (right traces) in both cells remained stable. (C) Time course of the normalized EPSP amplitude (averaged across both cells; n = 8 experiments) and the coupling coefficient (n = 11). (D) Population data for the ratio of normalized coupling coefficients after and before induction.

Since complex spike synchrony depends on both synaptic input and electrotonic coupling between olivary neurons (Marshall et al., 2007; Wise et al., 2010), it is important to know how the strength of chemical synapses is affected by protocols that modify gap junctional coupling. To test this, we assessed the chemical synaptic response (i.e., the evoked EPSP) along with electrical coupling strength. During the baseline and monitoring periods, we used a stimulus strength that would allow direct monitoring of the EPSP (without occlusion of the EPSP by olivary bursts). The induction protocol consisted of a steady-state depolarization (0–500 pA), with 100 synaptic stimuli at 1 Hz. The synaptic stimuli were paired with short depolarizing pulses (20 ms, 800 pA) to ensure reliable induction of burst spiking (Chorev et al., 2007; Khosrovani et al., 2007). As before, we found that the electrical coupling was depressed after induction (coupling reduced by 37% ± 7.5% of baseline, p < 0.01; n = 11 pairs; Figure 4). Since the EPSP was occasionally occluded by the low-threshold calcium spike or a rebound spike, our analysis was restricted to subthreshold synaptic responses (mean baseline EPSP size 6.4 ± 0.3 mV; n = 14 cells). We found that the strength of the chemical synapse in these cells did not vary significantly after induction (107% ± 7.8% of baseline; mean EPSP size after induction 6.1 ± 0.8 mV, p = 0.39, n = 17 cells). This indicates that plasticity induction is specific to electrical synapses and does not affect chemical synapses in the same cell, even though the chemical synapses have been used to induce the plasticity.

## Discussion

We have demonstrated that physiological activation of glutamatergic synapses triggers long-term depression of electrical coupling between inferior olive neurons while maintaining the strength of the chemical synapse. This provides a direct functional role for the precise anatomical arrangement of glutamatergic synaptic input and gap junction plaques in the synapse at the glomerulus that links multiple dendritic spines. The fact that chemical-electrical synapses have been shown to coexist throughout the mammalian nervous system, and the demonstration of similar intersynaptic plasticity mechanisms in the Mauthner cell of the goldfish (Yang et al., 1990; Pereda and Faber, 1996; Pereda et al., 1998; Smith and Pereda, 2003), suggests that this may represent a general principle. Our results provide a mechanism by which an extrinsic synaptic pathway can regulate the relative contribution of chemical and electrical synapses to the generation of synchronous patterns of activity, as well as an additional locus for long-term plasticity in the olivocerebellar circuit.

### Plasticity Mechanisms at Olivary Electrical Synapses

We show that depression of electrical coupling can be triggered by physiological patterns of synaptic input to olivary neurons involving low-frequency (1 Hz) stimulation of excitatory inputs, similar to the physiological frequency of firing of olivary neurons in awake animals (Armstrong and Rawson, 1979; Lang et al., 1999), but in contrast with plasticity of electrical coupling in the thalamus, which requires tetanic synaptic stimulation (Landisman and Connors, 2005). Higher-frequency stimulation (25 Hz) paired with 4 Hz olivary spikes did not induce changes in electrical coupling, although we cannot not rule out that other stimulation patterns may also trigger plasticity.

We demonstrate that induction of this form of long-term depression crucially depends on synaptic NMDA receptor activation and postsynaptic calcium elevations. Interestingly, these induction requirements are similar to those observed for long-term plasticity at chemical excitatory synapses throughout the brain (Bliss and Collingridge, 1993; Malenka and Bear, 2004). It is therefore surprising that the stimulated excitatory synapses that drove the electrical plasticity appeared to be resistant to change following the induction protocol. This indicates specificity of plasticity for the electrical synapses, in contrast to experiments in goldfish neurons (Yang et al., 1990; Cachope et al., 2007), and suggests that the olivary chemical synapses require different patterns of activity to induce plasticity.

We found that postsynaptic action potential bursts caused by intracellular current injections alone were not sufficient to cause plasticity, in contrast to a recent study in the thalamus (Haas et al., 2011). This suggests that calcium entry through voltage-gated calcium channels is insufficient to trigger the plasticity and that calcium entry through chemical synapses in proximity to the gap junctions could be playing an important role. Anatomical work has demonstrated that NMDA receptors are located within several microns of gap junctions at the olivary synapse (Hoge et al., 2011). Indeed, Hoge et al. (2011) already speculated that NMDA-receptor-mediated modulation of coupling could underlie the heterogeneous coupling coefficients found in the olive. Furthermore, it is known that CaMKII, which is activated by NMDA-receptor-mediated calcium entry (Lisman et al., 2002), is present close to Connexin 36 plaques in the inferior olive and that CaMKII and connexins can interact (Alev et al., 2008). Our experiments demonstrating that a CaMKII blocker prevents plasticity suggest that this is a candidate molecular effector, as recently demonstrated for NMDA-receptor-mediated activity-dependent plasticity of gap junctional coupling in the retina (Kothmann et al., 2012).

### Functional Implications

There is a growing awareness that plasticity in cerebellar circuits related to motor learning can take place at many sites, involving changes in both synaptic strength and/or intrinsic membrane currents (Hansel et al., 2001; Boyden et al., 2004). Our study provides an additional site for long-term changes in cerebellar circuits that involves changes in electrical coupling between a defined cell type.

How might this plasticity be functionally useful? Synchronous activity among groups of neighboring olivary neurons defines functional microzones that via climbing fiber input in turn drive synchronous activity of Purkinje cells. This microzonal organization is thought to act as a population code that represents distinct forms of sensory information. Synchrony in the olivocerebellar system is driven both by shared synchronous excitatory input to olivary neurons and electrical coupling. Our demonstration of plasticity of this coupling could provide the olivocerebellar system with a flexible way to remodel its microzonal architecture (Apps and Garwicz, 2005; Wise et al., 2010). While there is evidence that short-term modulation of olivary coupling can be driven by both glutamatergic and GABAergic inputs (Llinás, 1974; Lang, 2002; Jacobson et al., 2008; Hoge et al., 2011; Bazzigaluppi et al., 2012), which could provide dynamic regulation of microzone structure on the millisecond-to-second timescale, a long-term mechanism such as that shown here is required to sustain changes involved in cerebellar motor learning. In support of this model, there is evidence that long-term alterations in electrical coupling in the olive can impair cerebellar motor learning (Van Der Giessen et al., 2008). Since chemical synapses were not altered by our induction paradigm, glutamatergic synapses in the inferior olive may represent independent loci for shaping the patterns of synchrony underlying motor coordination. The long-term downregulation of electrical coupling by excitatory input may also represent a homeostatic mechanism for balancing activity during periods of synaptically driven synchrony.

## Experimental Procedures

### Slice Preparation

Transverse brain slices of the inferior olive (250 μm) were prepared from Sprague-Dawley rats (postnatal days 18–21) in accordance with national and institutional guidelines. Rats were anesthetized with isoflurane and subsequently decapitated. The brain was removed and submerged in ice-cold artificial cerebrospinal fluid (ACSF) bubbled with carbogen (95% O_2_, 5% CO_2_). The slicing ACSF contained 227 mM sucrose, 25 mM NaHCO_3_, 10 mM glucose, 5 mM KCl, 1.25 mM NaH_2_PO_4_, 0.5 mM CaCl_2_, and 3.5 mM MgCl_2_. The brain was cut parallel to the plane of slicing, and cyanoacrylate adhesive was used to fix the brain to the platform of a Leica VT-1200 S vibratome. Slices were transferred to a holding chamber and incubated for 30 min at 34°C in standard ACSF containing 125 mM NaCl, 25 mM NaHCO_3_, 25 mM glucose, 2.5 mM KCl, 1.25 mM NaH_2_PO_4_, 2 mM CaCl_2_, and 1 mM MgCl_2_ (pH 7.4). Slices were then kept at room temperature until they were transferred to a recording chamber and continuously superfused with oxygenated standard ACSF. All recordings were made at 33°C ± 1°C from slices that had been maintained at this temperature for at least 20 min (in light of the fact that gap junctional coupling is temperature sensitive [Payton et al., 1969]).

### Slice Electrophysiology

Patch pipettes were pulled from borosilicate glass on a PC-10 puller (Narishige) to a resistance of 5 MΩ for somatic recordings. The internal solution contained 130 mM KMeSO_4_, 7 mM KCl, 0.1 mM EGTA, 2 mM Na_2_ATP, 2 mM MgATP, and 0.3 mM Na_2_GTP (pH 7.2) (0.5% biocytin was included in some recordings). For voltage-clamp experiments, we used the same internal, except that KMeSO_4_ was replaced with 130 mM CsMeSO_3_. Olivary neurons in the principal and medial accessory olives were identified using infrared-DIC optics and somatic patch-clamp recordings were made under direct visual control (Stuart et al., 1993; Mathy et al., 2009), with most recordings being made from the principal olive. We selected somata within 50 μm of each other to maximize the probability of obtaining coupled cells (Devor and Yarom, 2002). Cells were rejected if their resting potential was depolarized to −40 mV. Coupling was assessed in current-clamp mode by injecting negative current pulses alternately (from −400 to −800 pA, every 4 to 6 s) in both cells. Bridge balance was monitored and corrected online and offline. For stimulation of synaptic inputs, a bipolar tungsten electrode (stimulus duration 50–100 μs) was placed in the white matter at the border of the olivary subnucleus from which the recording was obtained. During induction in current clamp, continuous depolarizing current was used to maintain a baseline membrane potential of 44 ± 1 mV, ensuring that cells fired with the physiological burst pattern seen in vivo and enhancing relief of magnesium block of NMDA receptors in experiments where spiking was not triggered by depolarizing current steps. Each slice was only used for one paired recording. To assess the strength of chemical synapses, we injected a 300 ms hyperpolarizing pulse (1 nA) in both cells and triggered an EPSP when the voltage had reached steady state. Biocytin processing was performed using standard techniques (Horikawa and Armstrong, 1988), with overnight paraformaldehyde (0.5%) fixation followed by conjugation with streptavidin-Alexa Fluor 488 (Invitrogen). Filled neurons were imaged using a 40× oil-immersion objective (NA 1.3) on a spinning-disk confocal microscope (Perkin-Elmer).

### Data Acquisition and Analysis

Data were low-pass filtered at 3–10 kHz and acquired at 20–100 kHz using an ITC-18 board (Instrutech) in conjunction with AxoGraph (AxoGraph Scientific) software. Analysis was carried out using custom-written software for MATLAB (MathWorks) and Igor Pro (Wavemetrics). The coupling coefficient was calculated as the ratio of the steady-state postjunctional and presynaptic voltage deflection caused by a prejunctional negative current injection (see Figure 1C). We report the coupling coefficient averaged between the two directions. Coupling conductance was estimated using the following formula (Hoge et al., 2011):

$$g_{c}={\left(R_{c}\right)}^{-1}={\left(\frac{R_{11}\times R_{22}-TR^{2}}{TR}\right)}^{-1}$$

where TR is the transfer resistance between the two cells averaged over both directions, and R11 and R22 are the input resistances of cell 1 and 2, respectively. About 30% of the recorded cells exhibited subthreshold oscillations, and in a minority of cells (<10%) the oscillations were sufficiently large that they precluded accurate measurement of coupling coefficients. For the chemical synapse, we used the peak of the EPSP to assess synapse strength. All data are reported as mean ± SEM unless otherwise indicated. Differences between groups were tested for statistical significance using Student’s two-tailed t test.

## References

[bib1] Alev C., Urschel S., Sonntag S., Zoidl G., Fort A.G., Höher T., Matsubara M., Willecke K., Spray D.C., Dermietzel R. (2008). The neuronal connexin36 interacts with and is phosphorylated by CaMKII in a way similar to CaMKII interaction with glutamate receptors. Proc. Natl. Acad. Sci. USA.

[bib2] Apps R., Garwicz M. (2005). Anatomical and physiological foundations of cerebellar information processing. Nat. Rev. Neurosci..

[bib3] Armstrong D.M., Rawson J.A. (1979). Activity patterns of cerebellar cortical neurones and climbing fibre afferents in the awake cat. J. Physiol..

[bib4] Bartos M., Vida I., Frotscher M., Meyer A., Monyer H., Geiger J.R., Jonas P. (2002). Fast synaptic inhibition promotes synchronized gamma oscillations in hippocampal interneuron networks. Proc. Natl. Acad. Sci. USA.

[bib5] Bazzigaluppi P., Ruigrok T., Saisan P., De Zeeuw C.I., de Jeu M. (2012). Properties of the nucleo-olivary pathway: an in vivo whole-cell patch clamp study. PLoS ONE.

[bib6] Beierlein M., Gibson J.R., Connors B.W. (2000). A network of electrically coupled interneurons drives synchronized inhibition in neocortex. Nat. Neurosci..

[bib7] Bennett M.V., Zukin R.S. (2004). Electrical coupling and neuronal synchronization in the Mammalian brain. Neuron.

[bib8] Blenkinsop T.A., Lang E.J. (2006). Block of inferior olive gap junctional coupling decreases Purkinje cell complex spike synchrony and rhythmicity. J. Neurosci..

[bib9] Bliss T.V., Collingridge G.L. (1993). A synaptic model of memory: long-term potentiation in the hippocampus. Nature.

[bib10] Boyden E.S., Katoh A., Raymond J.L. (2004). Cerebellum-dependent learning: the role of multiple plasticity mechanisms. Annu. Rev. Neurosci..

[bib11] Cachope R., Mackie K., Triller A., O’Brien J., Pereda A.E. (2007). Potentiation of electrical and chemical synaptic transmission mediated by endocannabinoids. Neuron.

[bib12] Chorev E., Yarom Y., Lampl I. (2007). Rhythmic episodes of subthreshold membrane potential oscillations in the rat inferior olive nuclei in vivo. J. Neurosci..

[bib13] Connors B.W., Long M.A. (2004). Electrical synapses in the mammalian brain. Annu. Rev. Neurosci..

[bib14] De Gruijl J.R., Bazzigaluppi P., de Jeu M.T., De Zeeuw C.I. (2012). Climbing fiber burst size and olivary sub-threshold oscillations in a network setting. PLoS Comput. Biol..

[bib15] de Zeeuw C.I., Holstege J.C., Ruigrok T.J., Voogd J. (1989). Ultrastructural study of the GABAergic, cerebellar, and mesodiencephalic innervation of the cat medial accessory olive: anterograde tracing combined with immunocytochemistry. J. Comp. Neurol..

[bib16] de Zeeuw C.I., Holstege J.C., Ruigrok T.J., Voogd J. (1990). Mesodiencephalic and cerebellar terminals terminate upon the same dendritic spines in the glomeruli of the cat and rat inferior olive: an ultrastructural study using a combination of [3H]leucine and wheat germ agglutinin coupled horseradish peroxidase anterograde tracing. Neuroscience.

[bib17] de Zeeuw C.I., Ruigrok T.J., Holstege J.C., Jansen H.G., Voogd J. (1990). Intracellular labeling of neurons in the medial accessory olive of the cat: II. Ultrastructure of dendritic spines and their GABAergic innervation. J. Comp. Neurol..

[bib18] De Zeeuw C.I., Simpson J.I., Hoogenraad C.C., Galjart N., Koekkoek S.K., Ruigrok T.J. (1998). Microcircuitry and function of the inferior olive. Trends Neurosci..

[bib19] Deans M.R., Gibson J.R., Sellitto C., Connors B.W., Paul D.L. (2001). Synchronous activity of inhibitory networks in neocortex requires electrical synapses containing connexin36. Neuron.

[bib20] Devor A., Yarom Y. (2002). Electrotonic coupling in the inferior olivary nucleus revealed by simultaneous double patch recordings. J. Neurophysiol..

[bib21] Gibson J.R., Beierlein M., Connors B.W. (1999). Two networks of electrically coupled inhibitory neurons in neocortex. Nature.

[bib22] Haas J.S., Zavala B., Landisman C.E. (2011). Activity-dependent long-term depression of electrical synapses. Science.

[bib23] Hansel C., Linden D.J., D’Angelo E. (2001). Beyond parallel fiber LTD: the diversity of synaptic and non-synaptic plasticity in the cerebellum. Nat. Neurosci..

[bib24] Hestrin S., Galarreta M. (2005). Electrical synapses define networks of neocortical GABAergic neurons. Trends Neurosci..

[bib25] Hoge G.J., Davidson K.G., Yasumura T., Castillo P.E., Rash J.E., Pereda A.E. (2011). The extent and strength of electrical coupling between inferior olivary neurons is heterogeneous. J. Neurophysiol..

[bib26] Horikawa K., Armstrong W.E. (1988). A versatile means of intracellular labeling: injection of biocytin and its detection with avidin conjugates. J. Neurosci. Methods.

[bib27] Jacobson G.A., Rokni D., Yarom Y. (2008). A model of the olivo-cerebellar system as a temporal pattern generator. Trends Neurosci..

[bib28] Khosrovani S., Van Der Giessen R.S., De Zeeuw C.I., De Jeu M.T. (2007). In vivo mouse inferior olive neurons exhibit heterogeneous subthreshold oscillations and spiking patterns. Proc. Natl. Acad. Sci. USA.

[bib29] Kothmann W.W., Massey S.C., O’Brien J. (2009). Dopamine-stimulated dephosphorylation of connexin 36 mediates AII amacrine cell uncoupling. J. Neurosci..

[bib30] Kothmann W.W., Trexler E.B., Whitaker C.M., Li W., Massey S.C., O’Brien J. (2012). Nonsynaptic NMDA receptors mediate activity-dependent plasticity of gap junctional coupling in the AII amacrine cell network. J. Neurosci..

[bib31] Landisman C.E., Connors B.W. (2005). Long-term modulation of electrical synapses in the mammalian thalamus. Science.

[bib32] Lang E.J. (2002). GABAergic and glutamatergic modulation of spontaneous and motor-cortex-evoked complex spike activity. J. Neurophysiol..

[bib33] Lang E.J., Sugihara I., Llinás R. (1996). GABAergic modulation of complex spike activity by the cerebellar nucleoolivary pathway in rat. J. Neurophysiol..

[bib34] Lang E.J., Sugihara I., Welsh J.P., Llinás R. (1999). Patterns of spontaneous purkinje cell complex spike activity in the awake rat. J. Neurosci..

[bib35] Leznik E., Llinás R. (2005). Role of gap junctions in synchronized neuronal oscillations in the inferior olive. J. Neurophysiol..

[bib36] Lisman J., Schulman H., Cline H. (2002). The molecular basis of CaMKII function in synaptic and behavioural memory. Nat. Rev. Neurosci..

[bib37] Llinás R. (1974). Eighteenth Bowditch lecture. Motor aspects of cerebellar control. Physiologist.

[bib38] Llinas R., Baker R., Sotelo C. (1974). Electrotonic coupling between neurons in cat inferior olive. J. Neurophysiol..

[bib39] Long M.A., Deans M.R., Paul D.L., Connors B.W. (2002). Rhythmicity without synchrony in the electrically uncoupled inferior olive. J. Neurosci..

[bib40] Malenka R.C., Bear M.F. (2004). LTP and LTD: an embarrassment of riches. Neuron.

[bib41] Manor Y., Rinzel J., Segev I., Yarom Y. (1997). Low-amplitude oscillations in the inferior olive: a model based on electrical coupling of neurons with heterogeneous channel densities. J. Neurophysiol..

[bib42] Marshall S.P., Lang E.J. (2009). Local changes in the excitability of the cerebellar cortex produce spatially restricted changes in complex spike synchrony. J. Neurosci..

[bib43] Marshall S.P., van der Giessen R.S., de Zeeuw C.I., Lang E.J. (2007). Altered olivocerebellar activity patterns in the connexin36 knockout mouse. Cerebellum.

[bib44] Mathy A., Ho S.S., Davie J.T., Duguid I.C., Clark B.A., Häusser M. (2009). Encoding of oscillations by axonal bursts in inferior olive neurons. Neuron.

[bib45] Mills S.L., Massey S.C. (1995). Differential properties of two gap junctional pathways made by AII amacrine cells. Nature.

[bib46] Payton B.W., Bennett M.V., Pappas G.D. (1969). Temperature-dependence of resistance at an electrotonic synapse. Science.

[bib47] Pereda A.E., Faber D.S. (1996). Activity-dependent short-term enhancement of intercellular coupling. J. Neurosci..

[bib48] Pereda A.E., Bell T.D., Chang B.H., Czernik A.J., Nairn A.C., Soderling T.R., Faber D.S. (1998). Ca^2+^/calmodulin-dependent kinase II mediates simultaneous enhancement of gap-junctional conductance and glutamatergic transmission. Proc. Natl. Acad. Sci. USA.

[bib49] Rash J.E., Dillman R.K., Bilhartz B.L., Duffy H.S., Whalen L.R., Yasumura T. (1996). Mixed synapses discovered and mapped throughout mammalian spinal cord. Proc. Natl. Acad. Sci. USA.

[bib50] Sloper J.J., Powell T.P. (1978). Gap junctions between dendrites and somata of neurons in the primate sensori-motor cortex. Proc. R. Soc. Lond. B Biol. Sci..

[bib51] Smith M., Pereda A.E. (2003). Chemical synaptic activity modulates nearby electrical synapses. Proc. Natl. Acad. Sci. USA.

[bib52] Sotelo C., Llinas R., Baker R. (1974). Structural study of inferior olivary nucleus of the cat: morphological correlates of electrotonic coupling. J. Neurophysiol..

[bib53] Stuart G.J., Dodt H.U., Sakmann B. (1993). Patch-clamp recordings from the soma and dendrites of neurons in brain slices using infrared video microscopy. Pflugers Arch..

[bib54] Tokumitsu H., Chijiwa T., Hagiwara M., Mizutani A., Terasawa M., Hidaka H. (1990). KN-62, 1-[N,O-bis(5-isoquinolinesulfonyl)-N-methyl-L-tyrosyl]-4-phenylpiperazi ne, a specific inhibitor of Ca^2+^/calmodulin-dependent protein kinase II. J. Biol. Chem..

[bib55] Van Der Giessen R.S., Koekkoek S.K., van Dorp S., De Gruijl J.R., Cupido A., Khosrovani S., Dortland B., Wellershaus K., Degen J., Deuchars J. (2008). Role of olivary electrical coupling in cerebellar motor learning. Neuron.

[bib56] Vervaeke K., Lorincz A., Gleeson P., Farinella M., Nusser Z., Silver R.A. (2010). Rapid desynchronization of an electrically coupled interneuron network with sparse excitatory synaptic input. Neuron.

[bib57] Wise A.K., Cerminara N.L., Marple-Horvat D.E., Apps R. (2010). Mechanisms of synchronous activity in cerebellar Purkinje cells. J. Physiol..

[bib58] Yang X.D., Korn H., Faber D.S. (1990). Long-term potentiation of electrotonic coupling at mixed synapses. Nature.

