# Supplementary material for: Synaptically Induced Long-Term Modulation of Electrical Coupling in the Inferior Olive
Source: Neuron. 2014 Mar 19;81(6):1290–6. doi: 10.1016/j.neuron.2014.01.005 (PMC3988996; doi:10.1016/j.neuron.2014.01.005)
Supplement: Document S1. Figures S1–S4 [file mmc1.pdf]

Neuron, Volume *81*

Supplemental Information

Synaptically Induced Long-Term Modulation of Electrical Coupling in the Inferior Olive

Alexandre Mathy, Beverley A. Clark, and Michael Häusser

Supplemental Information

Synaptically-induced long-term modulation of electrical coupling in the inferior olive

Alexandre Mathy, Beverley Clark and Michael Häusser

Inventory of Supplemental Information

Supplemental Figures:

Figure S1 shows that plasticity of gap junctional coupling can be measured either by estimating the gap junctional conductance, or directly using voltage clamp measurements.

Figure S2 shows that a high-frequency burst protocol does not produce depression of electrical coupling.

Figure S3 shows that plasticity of coupling does not require accompanying spikes during the induction protocol.

Figure S4 shows that plasticity induction can occur in the presence of GABAergic blockers, but it is blocked in the presence of a CaMKII blocker.

Supplemental Figures

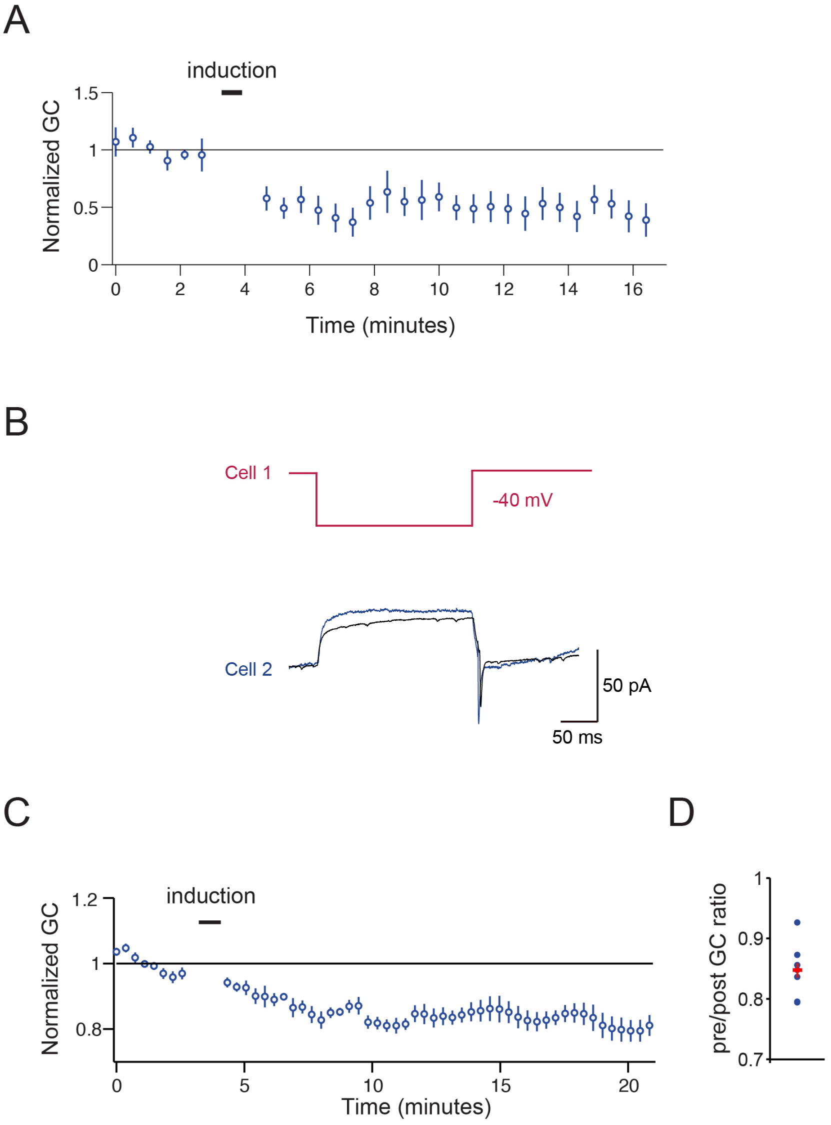

Figure S1. Plasticity of gap junctional coupling measured by estimating conductance and using voltage clamp. (Related to Figure 1)

A) Plasticity of coupling measured using the estimated gap junctional conductance. This figure contains data from the same cells as Figure 1 (n = 10 pairs). The coupling is significantly reduced after induction (48 ± 12% reduction; P < 1 x 10^{-4}). B) Cells were voltage-clamped at -55 mV and hyperpolarizing voltage steps (-40 mV) were injected alternately into both cells, with the coupling conductance used as a measure of coupling. Induction consisted of 100 synaptic stimuli paired with depolarization (10 ms, 5 mV) to allow burst firing in response to synaptic stimulation (colored traces: before induction, black traces after induction). Note that space clamp problems may result in an underestimate of changes in gap junctional conductance. C) Time course of changes in coupling following plasticity induction (n = 7 pairs). D) Population data of changes in coupling observed post-induction (P < 0.01).

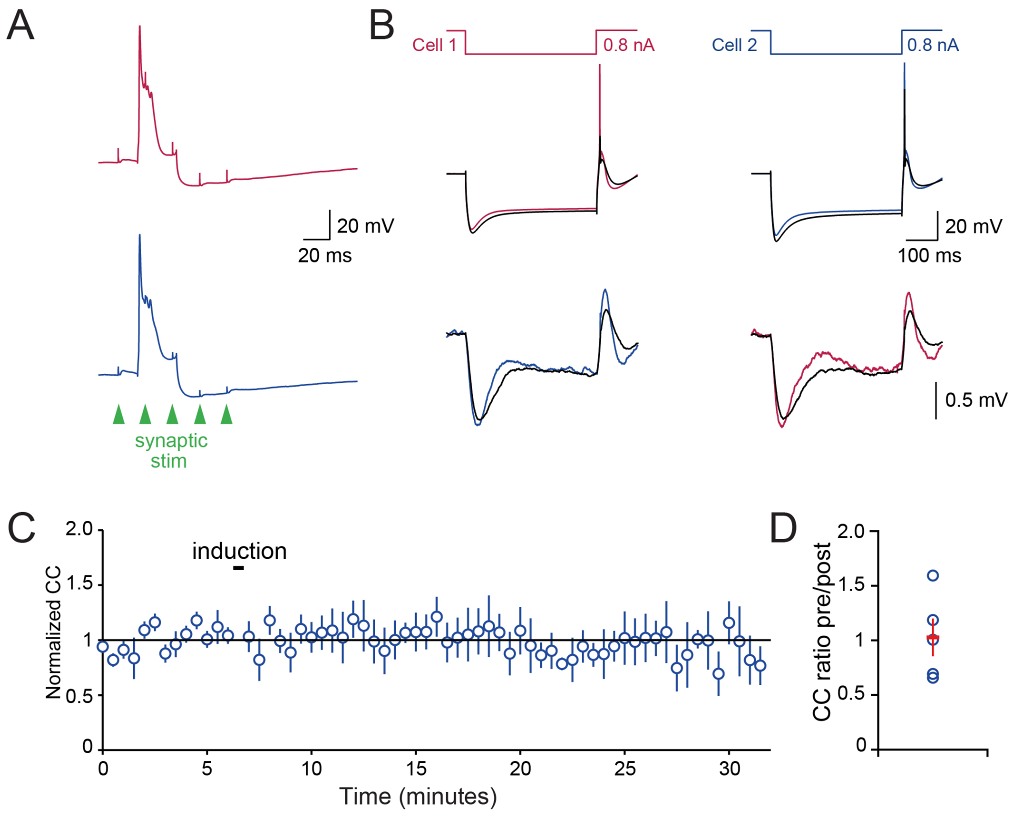

Figure S2. A burst induction protocol does not change coupling strength. (Related to Figure 2)

A) Induction protocol: Current-evoked olivary spikes paired with coincident bursts (25 Hz) of synaptic stimulation (100 pairings at 4 Hz). B) Example pair of olivary cells shows no change in junctional coupling (black traces: after induction; pre-induction traces in blue, cell 1, and red, cell 2). C) Normalized mean coupling coefficient for 5 cell pairs over the time course of the experiment. D) CC ratio for all cells (blue) together with mean ± SEM (red).

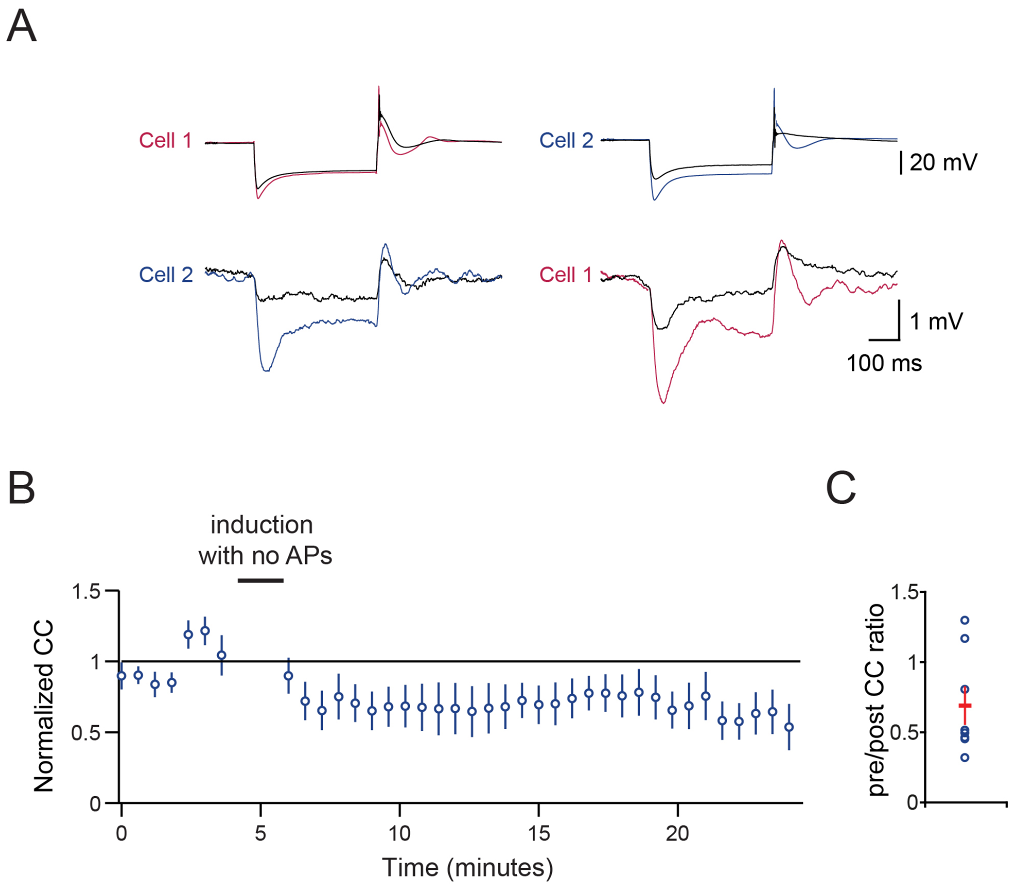

Figure S3. Plasticity induction with synaptic stimulation but with no accompanying spikes. (Related to Figure 2)

A) Example average responses (15 sweeps) to current injection before (coloured traces) and after (black traces) an induction protocol consisting of 50 synaptic stimuli at 1 Hz with no additional depolarizing pulses and accompanying action potentials. B) Time course of coupling (32 ± 13% depression after baseline, n = 7 pairs, P = 0.04). C) CC ratio for all cells (blue) together with mean ± SEM (red).

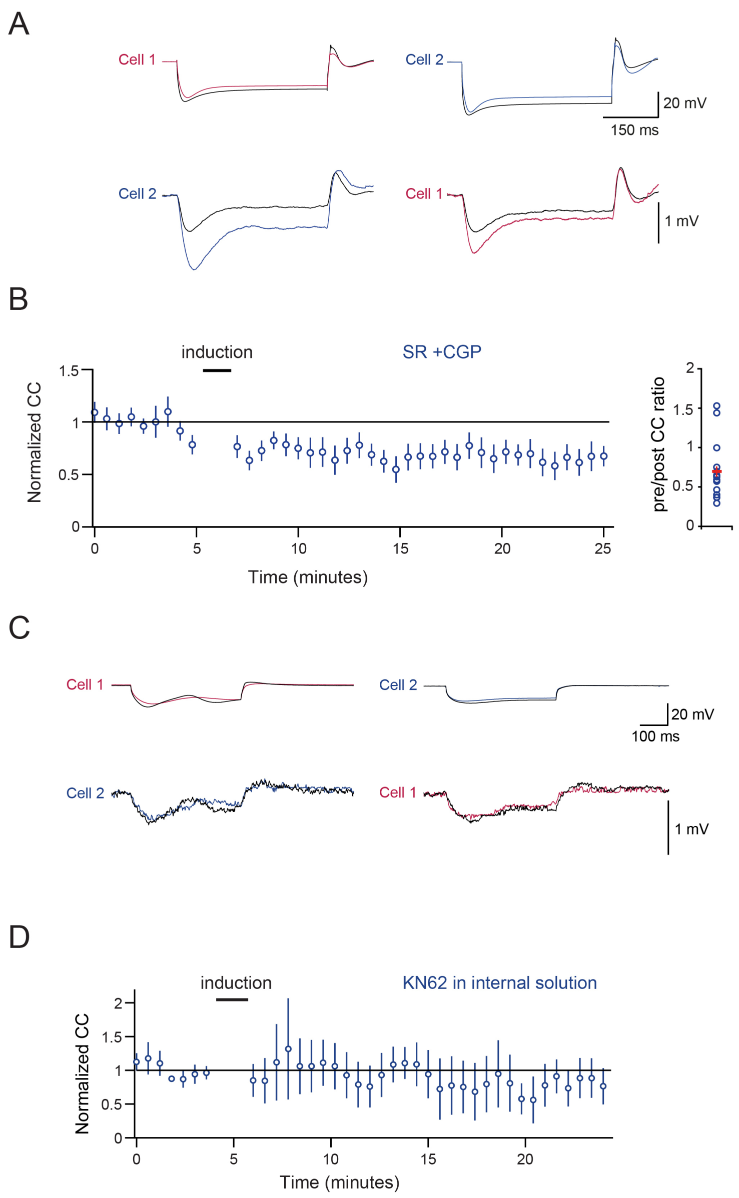

Figure S4. Plasticity induction proceeds in the presence of GABAergic blockers but is prevented by a CaMKII blocker. (Related to Figure 3)

A) Example average responses (50 sweeps) to current injection before (coloured traces) and after (black traces) induction carried out in the presence of bath-applied 10 μM SR95531 and 2 μM CGP55845 to block $GABA_A$ and $GABA_B$ receptors. Induction consisted of 100 synaptic stimuli paired with 20 ms, 800 pA depolarizations. B) Time course of mean normalized coupling coefficient (n=15 pairs). After induction, CC was reduced by 27.5 ± 9% P = 0.00031). C) Example average responses (15 sweeps) to current injection before (coloured traces) and after (black traces) an induction protocol carried out in presence of intracellular KN62 (10 μM). Induction consisted of 100 synaptic stimuli paired with 20 ms, 800 pA depolarizations. D) Time course of mean normalized coupling coefficient (89 ± 29% of baseline after induction; n = 4 pairs, P = 0.70).
